# A stakeholder-developed logic model to improve utilization of pharmacy-prescribed contraception in Utah

**DOI:** 10.1186/s43058-023-00503-6

**Published:** 2023-10-11

**Authors:** Rebecca G. Simmons, Jami Baayd, Casey Tak, David K. Turok, Sarah Elliott, Justin D. Smith

**Affiliations:** 1 University of Utah, 201 President’s Circle, Salt Lake City, UT USA; 2 Division of Family Planning, Health Educators, Leaders and Innovation Complex Suite #5050, University of Utah, 30 N. Mario Capecci Dr, Salt Lake City, UT 84112 USA

**Keywords:** Contraceptive access initiative, Contraception, Pharmacy contraceptive dispensing, Implementation science

## Abstract

**Background:**

Currently, 20 states in the USA have passed policies allowing pharmacists to prescribe short-acting hormonal contraception, including pills, patches, and vaginal rings. Yet, utilization of these services remains limited. The purpose of this study was to (a) assess barriers and facilitators of pharmacy contraceptive dispensing among contraceptive users, pharmacists, and healthcare providers in Utah and (b) adapt and propose an evidence-based contraceptive intervention in the pharmacy environment.

**Methods:**

We conducted 6 focus groups among contraceptive users, pharmacists, and healthcare providers assessing current barriers and facilitators to pharmacy prescribing. We coded transcripts of these focus groups to the Consolidated Framework for Implementation Research, Version 2.0 (CFIR) and characterized the findings based on the Expert Recommendations for Implementing Change (ERIC) Barrier-Busting tool. Based on the CFIR findings and ERIC strategies output, we adapted an existing evidence-based intervention (a contraceptive access initiative) to the Utah pharmacy environment. We then convened a pharmacy stakeholder meeting and presented elements of an Implementation Research Logic Model and obtained feedback. We coded this feedback to the CFIR framework to finalize an Implementation Research Logic Model for a proposed implementation approach to improving contraceptive prescribing.

**Results:**

Initial focus group responses clustered around specific implementation barriers including financial barriers (cost for patients, as well as lack of reimbursement for pharmacist’s time); lack of awareness of the service (on the part of patients, pharmacists, and health care providers); need for updated tools for contraceptive counseling and scheduling; and need for increased pharmacists education to conduct contraceptive counseling. Proposed adaptations to the existing contraceptive access intervention included development of a technology-based patient/pharmacist screener tool and a healthcare provider/pharmacist contraceptive referral network. Stakeholders identified pharmacist reimbursement as the top priority for improving utilization.

**Conclusions:**

Elements of contraceptive access initiatives mapped well as proposed implementation strategies to improving utilization of contraceptive prescribing in pharmacies.

Contributions to the literature

- Best practice implementation strategies commonly used in clinical contraceptive access initiatives can be adapted to address utilization of pharmacy-prescribed contraception.
- ERIC Barrier Busting tool outcomes can be mapped against existing evidence-based initiatives in order to identify gaps where additional strategies may be needed.
- Engaging community stakeholders in developing and improving the Implementation Science Research Logic Model *after* conducting initial barriers/facilitators research among the target population(s) can be a step-wise approach to identifying adaptations needed to implement strategies in specific communities.

## Background

More than 1.2 million people in the USA live in counties without a single health center offering the full range of contraceptive methods [1]. This contraceptive access gap means these people are less likely to obtain their preferred method of contraception, and they are less likely to use methods consistently and correctly [2]. Contraceptive initiatives throughout the USA have sought to improve access. A review of existing contraceptive access initiatives identified eight core implementation strategies that make up contraceptive access initiatives [3].

However, to date, contraceptive access initiatives have primarily focused on care delivered in healthcare clinics. While this work remains essential, additional access points beyond the traditional health setting are necessary to reduce unmet need among hard-to-reach populations. Some individuals live too far from clinics to access them [4] or are unable to take off work or find child care to visit clinics during daytime hours [5]. Novel approaches to expanding healthcare outside of the clinic setting include offering contraceptive care through online prescribing, via telemedicine, or through pharmacy contraceptive prescribing.

To date, pharmacist-prescribed hormonal contraception has been authorized in 20 states, including Utah [6]. Pharmacist-prescribed methods of hormonal contraception, including oral contraception, vaginal rings, and the contraceptive patch, are some of the most popular contraceptive methods available. Among the 47 million US women using a contraceptive method, pills, patches, and rings account for nearly a quarter (23%) of all methods used [7].

Pharmacy prescribing has the potential to improve access to preferred methods of contraception. Pharmacies are more prevalent than healthcare facilities, operate on extended hours, and typically do not require appointments ahead of time. These advantages of pharmacy-based care can mean increased access for historically underserved populations, such as clients who live in rural areas, are uninsured, or whose work schedules do not allow for typical healthcare access. In areas where pharmacy-based care is available, the clients availing themselves of contraceptive prescribing are typically younger, less educated, more likely to be uninsured, and report not having a primary care provider than individuals seeking care in traditional healthcare facilities [8]. Clients who use pharmacy prescribing report that pharmacy contraceptive services improve their ability to access contraception [9].

Despite the prospect of meaningfully improving contraceptive access, pharmacy prescribing remains both under-used among pharmacists and contraceptive clients. For example, in 2018, Utah passes a law allowing pharmacists to prescribe hormonal contraceptives, though the law does not allow pharmacists to bill third-party payors, including Medicaid, for those services. A secret shopper study conducted in Utah in 2019 found that only 27% of pharmacies were providing contraceptive dispensing, and most were only offering combined oral contraceptives (despite being allowed to prescribe progestin-only pills, vaginal rings, and contraceptive patches) [10]. On the demand side, studies of uptake among potential clients consistently show that few people are accessing contraceptive methods this way. A study conducted in Oregon, which allows Medicaid reimbursement for pharmacy-prescribed methods, found that fewer than 2% of Medicaid contraceptive claims were pharmacy-based [11].

The lack of implementation success to date even in settings with supportive policies implies that it is insufficient to simply pass legislation and expect substantive change without additional strategies targeted at multilevel barriers to implementation. Yet, an understanding of which implementation barriers are most hindering uptake remains limited. If barriers to pharmacy contraceptive prescribing are similar to barriers that have historically limited contraceptive access in healthcare organizations, existing evidence-based initiatives with specific strategies could be employed to improve contraceptive access in these novel settings. The purpose of this study was to identify barriers to pharmacy contraceptive prescribing in Utah and assess if and how the components of traditional contraceptive access initiatives might be successfully adapted to pharmacy settings.

## Methods

We sought to answer two questions in this study: (1) what are the barriers and facilitators to implementing pharmacy contraceptive prescribing?; and (2) what would a pharmacy contraceptive access initiative look like in Utah given context-specific barriers and facilities? To this end, we designed and conducted the study using a pragmatic analytic approach, [12] using three interrelated and complementary implementation tools: the Consolidated Framework for Implementation Research (CFIR) Version 2.0, [13] the Expert Recommendations for Implementing Change Barrier-Busting Tool (ERIC), [14] and the Implementation Research Logic Model [15]. These tools worked synergistically with one another to help us answer our two study questions. An overview of the tools and their use in this study is provided in Table 1. Broadly, we (1) conducted focus groups with clinic-based providers, pharmacists, and contraceptive users to identify barriers and facilitators for pharmacy-prescribed contraception; (2) analyzed the focus group findings using the CFIR; (3) input CFIR findings into the ERIC Barrier Busting tool; (4) mapped ERIC constructs against the main evidence-based strategies of contraceptive access initiatives to develop an initial IRLM [15]; (5) hosted a meeting of stakeholders to review and provide feedback on the components of the IRLM, particularly the proposed implementation strategy package; and (6) revised the IRLM and strategies to reflect feedback from stakeholders. The study was initiated and designed by the first author (RS), who has expertise in qualitative and quantitative research, with additional training in implementation science. The first author and co-authors (JB, SE, DKT) have conducted and overseen large-scale contraceptive access projects in Utah since 2015, with the goal of improving high-quality, person-centered contraceptive services in the state. Co-author JDS is the lead developer of the IRLM and has used it with community partners for other implementation projects [16, 17]. The current study is an initial step in planning an implementation project to improve contraceptive prescribing among pharmacists. This study was approved by the University of Utah Institutional Review Board (IRB # 00152100).

**Table 1:** Overview of study implementation questions and the models/frameworks used to answer them

**Implementation question**	What are the specific barriers and facilitators of pharmacy contraceptive prescribing in Utah?	What are the recommended implementation strategies needed to address identified barriers?	What does pharmacy-based contraceptive access project aimed at reducing these barriers look like?
**Implementation approach**	• Focus groups with pharmacists, providers, contraceptive users • Stakeholder input	• CFIR results • Additional stakeholder input	CFIR results + ERIC strategies + final stakeholder input
**Implementation research method and model framework**	Consolidated Framework for Implementation Research 2.0	ERIC Barrier Busting Tool	Implementation Science Research Logic Model

### Initial focus groups

Our team conducted focus groups with three groups: contraceptive users, pharmacists, and healthcare providers. We developed interview guides for each group, aimed at understanding participant perspectives of barriers and facilitators to current pharmacy contraceptive prescribing in Utah. The discussion guides included questions such as *are you aware that pharmacists in Utah can prescribe contraception*;* what do you see as the advantages and disadvantages to pharmacy prescribed contraception*;* (for providers and pharmacists) how would you like to see pharmacists and providers working together to support contraceptive access*; and* (for pharmacists) what are the barriers to providing contraception in your pharmacies (and what solutions have you found to these barriers)?*

Eligibility for the contraceptive user focus groups included current residency in Utah, being of reproductive age (18–50), speaking either English or Spanish, and having a history of contraceptive use. Eligibility for the pharmacist focus groups included being a current practicing pharmacist in Utah. Eligibility for inclusion in the provider focus groups included being a current practicing healthcare provider (i.e., doctor of osteopathy, nurse practitioner, physician’s assistant, medical doctor).

Support with participant recruitment and conducting the focus groups was provided by the University of Utah’s Community Collaboration and Engagement Team (CCET). Participants for the different focus groups were recruited through convenience sampling, including word-of-mouth and flyers that are distributed in various relevant organizations, such as clinics, social media sites, and other community organizations. Interested individuals scanned the QR code provided in the recruitment materials and were screened for initial eligibility. Upon meeting initial eligibility, potential participants were then contacted by a member of the Engagement team to review the study, provide consent documents, and ensure each individual fully met inclusion/exclusion criteria. Consenting participants were invited to attend focus groups, which were hosted and facilitated by the CCET over Zoom from wherever they were located. At least one member of the study team (either RS or SE) was present for each focus group to introduce themselves, explain the study purpose to participants, answer any participant questions, and ask pertinent follow-up questions. One of the contraceptive user focus groups was held in Spanish by a Spanish-speaking CCET facilitator. All focus group conversations were recorded and subsequently transcribed by a professional transcription service. The Spanish-speaking focus group was first transcribed into Spanish and subsequently translated into English by a professional translation service.

### Focus group analysis

All transcripts were uploaded to MAXQDA [18] for analyses. Transcripts were then deductively coded to CFIR, Version 2.0. 13 This approach was selected because it specifically identifies potential barriers and facilitators to implementation of healthcare interventions. Three members of the study team (RS, JB, CT) collaboratively coded two initial transcripts to discuss the coding structure, establish agreement, and tailor the CFIR codebook as needed. All subsequent interviews were individually coded by the three study team members. Upon completion of initial coding, the study team met to discuss and resolve any outstanding coding questions. CFIR constructs identified in the transcripts were then uploaded to the ERIC Barrier-Busting tool [16]. ERIC strategies and CFIR constructs were then mapped onto the existing best practice strategies for conducting statewide contraceptive initiatives. Strategies that did not map onto the existing contraceptive initiative were proposed as potential pharmacy-specific adaptations to a contraceptive initiative.

### Stakeholder convening

Prior to convening our stakeholder meeting, we used the IRLM to develop an initial logic model for a pharmacy contraceptive access initiative [14]. The IRLM incorporated CFIR findings from the focus groups, suggested strategies from the ERIC tool, and the key components of contraceptive initiatives as identified by prior studies, plus proposed modifications to the existing components. Upon completion of the draft IRLM, our study team recruited community stakeholders via email to participate in a 2-h in-person meeting on October 7, 2022, to review the proposed intervention and provide feedback around its acceptability and feasibility. Community stakeholders were invited if they were involved in pharmacy contraceptive dispensing in any capacity and included commercial pharmacies, healthcare pharmacies, members of the Utah Board of Pharmacy, faculty from the University of Utah College of Pharmacy, members of the Utah Department of Maternal and Child Health, the Utah Medicaid office, and the state Department of Health and Human Services. Individuals were recruited through snowball sampling and encouraged to invite additional people they felt would be relevant to the conversation.

During the meeting, our study team (RS and SE) presented findings from the focus groups, highlighting key barriers and facilitators. After presenting our findings, we asked the stakeholders to identify missing barriers or facilitators. Next, we proposed the adapted contraceptive initiative intervention package as detailed in the IRLM and facilitated a discussion about the acceptability, feasibility, and potential barriers to each element of the proposed intervention. Stakeholders voted on elements of the implementation strategy package that they felt were most pressing. Three members of the study team wrote field notes and memos to capture the full conversation during the meeting.

### Stakeholder analysis and finalizing IRLM and implementation strategy package

We framed our stakeholder analysis and IRLM modifications using guidance from Knapp et al. (2022) [17]. One member of the study team (JB) consolidated the field notes and coded them in MAXQDA in accordance with CFIR 2.0. Members of the study team then met to review findings and modify any elements of the existing IRLM in accordance with stakeholder feedback. Adapted elements of the intervention were then provided back to the attendees from the stakeholder convening to solicit any final feedback.

## Results

### Initial focus group results

We completed a total of six focus groups: two groups with pharmacists, two with contraceptive clients, and two with healthcare providers. Each of the groups lasted between 60 and 90 min. A total of 6 pharmacists, 15 contraceptive clients, and 7 healthcare providers participated in the focus groups. Among the pharmacists, four were doctors of pharmacy (PharmDs) and two were pharmacy directors. Among contraceptive clients, all were between ages 23 and 36, all identified as women, 8 identified as Hispanic/Latina, and there was an even split between rural (*n* = 5), urban (*n* = 5), and suburban (*n* = 5) residence. Among providers, there was some diversity in licensure, with two medical doctors (MDs), two generalist nurse practitioners (MSNs), 2 Doctors of Nursing Practice (DNPs), and 1 specialty nurse practitioner (APRN).

Our focus group findings clustered on 10 CFIR2.0 constructs. Five of the constructs were found within the outer setting domain, suggesting many of the challenges to implementing are related to challenges outside the pharmacy setting, including issues of financing (e.g., lack of pharmacist reimbursement mechanisms for service provision), integration with existing policies/laws, challenges in partnerships and connections, and aspects of local conditions and attitudes (e.g., healthcare provider concerns about pharmacy contraceptive clients not returning to healthcare organizations for other health services). An overview of each construct with representative quotes can be found in Table 2.

**Table 2:** Pharmacy contraceptive prescribing focus groups mapped to CFIR

CFIR construct	Key concepts within the construct	Representative quote(s)
**Innovation domain**		
Complexity	• The risk involved in contraception is sometimes overrepresented • It can be overwhelming for pharmacists to learn the contraceptive counseling process • Concern about liability if prescribed for a patient who is not a good fit for a certain method	*Yes, there are definite risks to birth control; there are risks to a lot of things that are sitting on the counter [...] It just feels birth control’s been targeted in a way that maybe Tylenol hasn’t, you know. And how many people overdose on Tylenol as a percentage of people taking Tylenol versus like people who end up with a stroke out of the birth control pills?* *-*Provider
Adaptability	• The state-required process to become eligible can be cumbersome	*Having patients be empowered to screen themselves, understand where they’re at themselves, is overall better for health care in general just because they themselves are more educated on their conditions moving in so that we’re more fit to help them* -Pharmacist
**Outer setting domain**		
Financing	• Need reimbursement for pharmacists’ counseling time • Uninsured patients cannot afford some options	*As far as some barriers to providing contraceptive care [...] The majority of the patients I did prescribe birth control for were without insurance or were between insurances, so that immediately reduced the availability of certain ones for instance, NuvaRing, patches, things like that, they definitely go up in cost* -Pharmacist
Policies and laws	• Required visits to providers are a barrier • Parental consent laws limit access for clients under 18 • State requirements for pharmacist prescribing take time but are worthwhile	*The birth control still works for me, so going to the office [to comply with the law requiring yearly physician follow-up] and paying that copay is a little bit of a barrier. Pretty soon, when I won’t have health insurance anymore, then it’ll be a complete stop. I won’t even be able to go to a provider and get that prescription* -Client *As far as the how easy it is in law for us to do, it is a little bit tricky. We have to maintain the two hours of continuing education. You have to enroll, but it was super nice that there’s those forms, like the questionnaires that are already built out and readily available for us. I feel like it’s somewhere in the middle between easy and hard to do, but it does take a little bit of effort* -Pharmacist
Partnerships and connections	• Desire for integrated EHRs for provider/pharmacist communication • Desire to build trust between providers and pharmacists	*Yeah. I know in our practice when we collaborate with our physicians [...] the physicians really do like the services that we bring to the table. In fact, they go out of their way to make sure that they’re reaching out to us on different things when they know that they can trust us and that we are there to help them out. I think ultimately that kind of relationship can be developed even within the retail setting and the physician providers, too* -Pharmacist
Local conditions	• Students have trouble accessing contraception • Lack of sex ed • Lack of transportation • Desire for education about contraceptive options • Desire for client-led decision making • Need for better advertisement of pharmacy as an access point for contraception	*Barriers come in many forms. Cost is often one, but even with that barrier being taken care of, at the end of the day, the big thing was if people don’t know about it, they can’t utilize it. I think that’s the first big barrier we need to overcome for pharmacy providing contraception* -Client
Local attitudes	• Clients appreciate the convenience of pharmacy-prescribing • Some do not view their pharmacies as a place for education • Some clients prefer pharmacists to providers for contraceptive conversations • Pharmacists are limited in providing the full range of methods • Some providers worry lost opportunity to address other women’s health needs	*I think that this is a wonderful idea just for people that fit in my demographic. You know, I’ve been on birth control my entire life. I’m friggin’ busy. I don’t have time to remember to go to the doctor. Sometimes I’m showing up to get milk. I’m like, oh crap, I’m out of my blood pressure medicine, too. I think this would be potentially great for just 30 -, and 40-year-old women that are friggin’ busy. And it’s smooth sailing. We’ve been on the same pill for that last decade. I just need a refill* -Client *Then, I think I’d also be more comfortable talking to a pharmacist because it’s their job to administer different kinds of medication and I feel like if they see so many different things that they probably would not be very judgmental* -Client
**Inner setting domain**		
Access to knowledge and information	• Pharmacists unaware of ability to prescribe • Primary care providers unaware of which pharmacies offer this service • Pharmacists find the continuing education modules helpful but would like more information • Medical Eligibility Criteria is helpful	*We have not implemented this program either at our [pharmacy]. Part of it is just even the information for pharmacies to know that this is out there and available and what training and resources are out there to help them implement this kind of a program. I think that would go a long way to getting more pharmacies up and on board with it* -Pharmacist
Culture > recipient-centeredness	• Clients feel pharmacists might be more person-centered than providers • Pharmacists and providers want to improve access for clients • Pharmacists are concerned about client privacy	*And patients will be waiting for months on end to get in [to see provider] just for a birth control visit. That’s absurd. They should have access birth control much faster than that* -Provider *I think that our whole purpose is doing what’s in the best interest of our patient and expanding care. I think we can all agree that this [pharmacy prescribing] is a fantastic idea. It’s just getting all the pieces in place so that it’s safe for our patients and that it’s a smooth process when they go in* -Provider
Structural characteristics	• Need for private counseling space • Some pharmacy cultures/policies do not allow time for counseling sessions • Provision of vaccines in pharmacies created systems that can be used for other services	*I don’t usually bring this one up, but I do think that we continually talk about time being a barrier* [...]. *The way that our model works, we will allow labor to be earned like it would be for a prescription, but—for the time of a visit for something like this, but until you get to a level of services that allows you to then add an extra pharmacist so that there’s two pharmacists on some days where you can schedule appointments during overlap or something. You’ve got to get to a level of services that—high level that allows for that* -Pharmacist
**Individual domain**		
Innovation recipients (patient need)	• Lack of healthcare access • Complicated patient needs Affordability • Desire for a female pharmacist • Lack of awareness of pharmacy prescribing • Need for walk-in appointments • Desire for privacy • Need for multiple languages • Shame around contraception	*I like the idea of the drugstore. First because, when I was younger, I used to be ashamed to visit the doctor; whereas in the drugstore, it’s a lot easier to go, talk and be seen* -Client *So, for me, an ideal situation would be that the person spoke Spanish and was able to understand me. In general, I have realized that we, Latin people, tend to speak a lot; so, it would be very good if they could give you comprehensive information. You would feel good with that* -Client *I think [ideal situation] would be without an appointment. It’s the easiest and the most practical. They are there to assist us and to solve our questions in a much faster way than to make an appointment with a gynecologist. We know they are there for us in a much faster way* -Client

The table includes all CFIR constructs to which at least 10 excerpts were coded, with the exception of the Innovation domain, which each had 7 in the subdomains

### Mapping of CFIR constructs and ERIC strategies with contraceptive access initiative strategies

The initial CFIR2.0 findings uploaded into the ERIC tool yielded 23 *most recommended* strategies. These are mapped to the eight core strategies of contraceptive access initiatives in Table 3. No main ERIC strategies mapped to provision of grants for equipment and supplies. Five ERIC strategies did not map to any core elements of contraceptive initiatives.

**Table 3:** Mapping ERIC strategies onto core strategies of contraceptive access initiatives

Core strategies of contraceptive access initiatives	ERIC recommended strategies emerging from focus groups
Training and education for healthcare teams	Create a learning collaborative
	Conduct educational meetings
	Work with educational institutions
	Shadow other experts
Ongoing technical assistance	Centralize technical assistance
	Facilitation
Provision of low-/no-cost contraception to clients	Alter patient/consumer fees
Grants for equipment and supplies	
Public awareness campaign	Start a dissemination organization
Quality improvement/data monitoring and evaluation	Conduct local needs assessment
	Assess for readiness and identify barriers and facilitators
Multistakeholder partnerships with public/private entities	Identify and prepare champions
	Build a coalition
	Conduct local consensus discussions
	Capture and share local knowledge
	Inform local opinion leaders
	Identify and prepare champions
Policy change to improve access to contraception	Alter incentive/allowance structures
	Place innovation on fee for service lists/formularies
	Revise professional roles
*Unmatched strategies*	Promote adaptability
	Develop disincentives
	Create new clinical teams
	Change record systems

### Identifying additional strategies and developing the initial IRLM

Focus group participants identified areas that did not necessarily align with existing evidence-based strategies. For example, pharmacists noted that the existing state-required contraceptive screener tool was a barrier to implementation. One pharmacist noted, “One thing I can think of is using a little bit of automation. For myself, using the quick prescreen, that totally could be in an automated fashion. If we routed it so that it was referred to an online form in which it does the prescreen to a certain extent before gaining consent moving forward in the process with the understanding that, at the end of the day, it’s—they still might not be qualified to receive it. That would save on a lot of the time factor.”

Pharmacists and healthcare providers also touched upon the general lack of integration between the two professions in provision of primary care services. Healthcare providers identified hesitancy within the profession to have pharmacists provide contraceptive services, noting concerns that patients would then be disincentivized to return to a healthcare clinic for other primary care services, such as Pap screenings, or complicated contraceptive care. One provider explained, “I was gonna say my initial response; I remember when the bill passed, and my response was, oh, wait a minute, I did all this training to figure out who could have—it was honestly a little turfdom, if you will, of like, I did all this training, and do pharmacists really know how to do this? And can doing an online module—which I think they were required to do some online module to be able to do it—it was like, is that really gonna replicate years of training?”

While noting those concerns, many providers and pharmacists also identified the untapped potential to improve patient contraceptive care through collaboration. A pharmacist participant noted, “I think sometimes it just takes one person starting it. Once the doc sees that you’re willing to refer your IUD cases to them, they become a little bit more open to the idea that you’re helping bridge a gap that they’re not able to meet. Clearly, at each pharmacy, depending on where you’re at, you have your X number of doctors that are your close, your local, your main ones that you do the majority of your scripts from. If you focus on making your collaboration with them, they could see you as a benefit to their community of patients and not a problem.”

Finally, pharmacist participants also noted the critical importance of pharmacy billing and reimbursement in improving pharmacy contraceptive prescribing potential, both with respect to patient affordability and around service sustainability. One pharmacist stated, “Just the ability to do anything it requires that there’s fair compensation for the time of the health care providers that are doing it. Just the model in pharmacy that I’ve been working in for over 20 years is just messed up. [Laughter] We need to be paid for the things that we do other than just the prescription that gets dispensed, and charging cash for patients is hard.”

Based on the mapping exercise and focus group responses, we identified three additional strategies to supplement the existing core contraceptive access initiative strategies. These included (1) creation of a bidirectional pharmacist/provider network (shadowing other experts; creating new clinical teams); (2) improving the existing contraceptive screener tool (change record systems); and (3) providing pharmacist reimbursement for contraceptive services (e.g., the counseling encounter). These elements were added to the eight core elements for a total of eleven implementation strategies (Table 4).

**Table 4:**
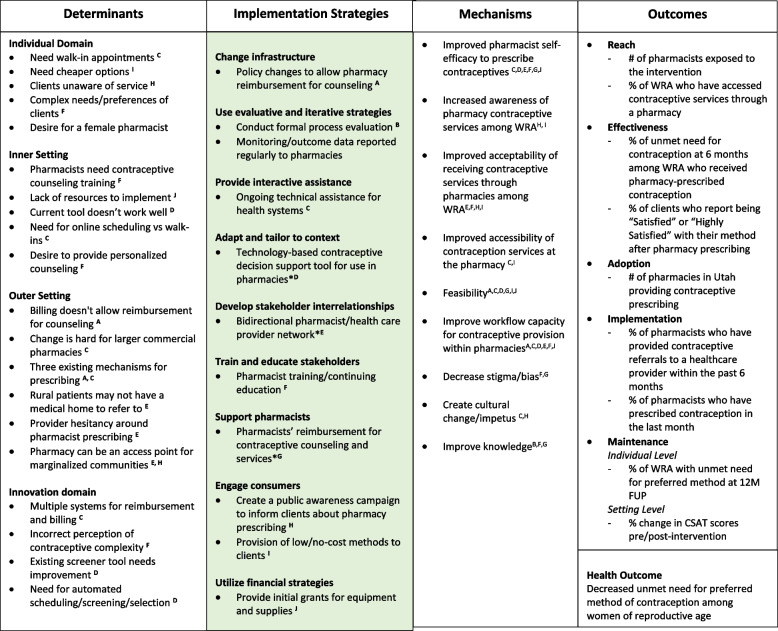
Revised Implementation Science Research Logic Model

Superscript letters = determinants and mechanisms are matched to the strategies identified

### Stakeholder convening

A total of 12 pharmacy stakeholders attended the October 7th meeting. Attendees reviewed the initial focus group findings and were asked if any major barriers were missing. While participants did not identify any elements that were missing, they did feel the context surrounding several barriers was incomplete. For example, participants noted that pharmacy reimbursement challenges were influenced by which prescribing mechanism a particular pharmacy organization was using. Since Utah allows contraceptive prescribing under collaborative practice agreements, an existing standing order, and a direct prescribing law, different pharmacies have different challenges with pharmacist reimbursement according to which mechanism they are using.

After discussing focus group findings, the research team presented the proposed intervention elements, including both the core elements of standard contraceptive access initiatives and the three additional strategies identified through the preliminary research. Stakeholders provided additional context for each of the elements. For example, when discussing provision of low/no-cost methods to contraceptive clients, stakeholders identified the question of whether the pharmacist is providing a single cycle of contraception or multi-cycles of contraception (best practice) and how this could influence costs. After discussion of all elements, stakeholders were asked to identify which intervention strategy they thought was most pressing. All attendees identified pharmacist reimbursement as the most challenging and most important barrier to address.

### Updated IRLM

After coding field notes and memos from the stakeholder convening, we updated the IRLM to reflect both the additional complexity of CFIR2.0 constructs and mechanisms of change. The revised IRLM can be found as Table 4. The final presentation to stakeholders (which occurred both in-person and electronically, depending on the attendee’s preference) did not result in any substantive changes to the adapted IRLM.

## Discussion

Our study identified several barriers to implementation of pharmacy prescribing in Utah. Our findings largely overlap with the findings of a systematic review of implementation barriers to pharmacy prescribing; all barriers identified in the systematic review were also identified in our state-specific assessment, including major barriers such as pharmacist reimbursement, lack of patient awareness, lack of training, and corporate policies that inhibit utilization [19]. Importantly, however, our assessment identified some additional barriers to prescribing that were either not described in the systematic review (e.g., incorrect perceptions of contraceptive complexity among both pharmacists and healthcare providers) or which were identified in the review as facilitators but were barriers in our study. For example, collaborating with other professionals was seen as a facilitator in the systematic review, while our study showed that some existing tensions between healthcare providers and pharmacists around the role of the pharmacist in primary care can be a barrier to comprehensive patient care if not specifically addressed. Similarly, the review noted that the existence of a contraceptive prescribing protocol was a facilitator to implementation, while participants in our study noted that the current tool (which, in Utah, is paper-based and must be taken by the client onsite and then used by the pharmacist to apply to a specific eligibility algorithm) was a barrier. These differential findings highlight the importance of assessing the setting context when developing and tailoring implementation strategies within this context.

The strategies identified through our inputs into the ERIC tool mostly mapped onto evidence-based strategies of community contraceptive access initiatives. Neither focus group participants nor stakeholders identified a need for grants to purchase supplies or support contraceptive prescribing, which is a key strategy within contraceptive access initiatives. This may be representative of the differences between provision of comprehensive contraception, which typically requires specific products, machinery, or supplies, like an autoclave in order to offer intrauterine devices, versus the more straightforward provision of pre-packaged contraceptive methods like pills. Or, it may simply be indicative that to date, fewer contraceptive implementation interventions have occurred in pharmacies, and offerings like grants to provide blood pressure machines or support additional staff time are simply unfamiliar to nontraditional stakeholders. Specific evaluative mechanisms to assess this will likely be important to inform future pharmacy-based contraceptive initiatives.

Our study also identified three additional strategies not traditionally applied in contraceptive access initiatives. Interestingly, each of these strategies mirrors identified facilitators in the systematic review of pharmacy contraceptive prescribing implementation [19]. For example, the goal of developing a pharmacist-healthcare provider contraceptive network is specifically to improve collaboration with other healthcare professionals and integrate pharmacists more broadly into the primary care network, thereby improving both pharmacist self-efficacy and patient referral pathways. These three additional strategies have yet to be tested as part of the existing bundle of implementation strategies for contraceptive initiatives, as they appear unique to pharmacy settings. These additional strategies, and their hypothesized mechanisms of impact, warrant additional study to determine if they should be added as best practices to future pharmacy-based contraceptive initiatives.

Pharmacist reimbursement for providing contraceptive services (i.e., counseling) was identified as a key barrier to improving prescribing implementation in our qualitative interviews and as *the* main barrier to uptake in our conversations with stakeholders. Since pharmacists were not identified as healthcare providers in the Social Security Act of 1935, their ability to bill and be reimbursed for healthcare services remains a challenge. This oversight is increasingly cumbersome as pharmacists continue to take on critical roles in primary care provision—in contraception as well as through provision of vaccines, smoking cessation programs, or opioid addiction treatment. As incorporating these services moves pharmacists further into primary care provision, this shift will require changes in the healthcare systems. Ideally, the policies and systems will change at the federal level, through the Centers for Medicare and Medicaid Services (CMS), to allow pharmacists to be considered healthcare providers. This would solve many of the current challenges with reimbursement.

Absent a federal change, some states are taking different approaches to addressing this issue—some through pharmacist reimbursement legislation mandating Medicaid or commercial insurance reimbursement for hormonal contraception prescribing (e.g., California and Oregon), some through creative interpretation of pharmacy prescribing, and some through the creation of Current Procedural Terminology (CPT) codes that include counseling services. Utah currently does not have legislative allowances for pharmacy reimbursement for contraceptive service provision. Thus, the costs for providing family planning services are passed along to the contraceptive client, often costing more than $35 per visit, in addition to the costs of the contraceptive product. Ultimately, the lack of pharmacist reimbursement appears to create a negative feedback loop where the service cost of pharmacy prescribing becomes a barrier to clients, who then do not seek access to these services, which then reduces pharmacy contraceptive demand, ultimately leading to fewer contraceptive visits, thereby decreasing pharmacist self-efficacy around contraceptive prescribing and reduced willingness to offer these services. This problem persists even among states that have passed legislative changes to allow pharmacists to bill for Medicaid reimbursement, yet still see low levels of utilization. Ultimately, both policy change and specific implementation strategies are critical to improving the acceptability of pharmacist reimbursement for services among all insurers.

This study assessed critical barriers to utilization in a single state and developed a proposed pharmacy-based contraceptive access initiative with stakeholders identifying specific implementation strategies to improve utilization. This pharmacy-based contraceptive access initiative provides a roadmap for implementers in the state looking to improve pharmacy prescribing. Furthermore, the methods described could be used in other states to plan for context-specific pharmacy-based contraceptive access initiatives and implementation research projects. Our generative process in creating the logic model could be mirrored by other states looking to address low utilization of pharmacy prescribing. Future research will be needed to test the strategies proposed to determine if they produce the hypothesized mechanisms of impact and corresponding outcomes outlined in the logic model.

## Conclusions

Pharmacy contraceptive prescribing is a promising approach to reducing contraceptive access deserts. Pharmacists are supportive, contraceptive clients are interested, and legislative policy is in place; yet, without specific application of key implementation strategies, this offering remains underutilized and ultimately limited in impact. Contraceptive access initiatives provide a basis for improving pharmacy contraceptive prescribing; however, adaptations and new strategies are needed to account for the different policy and regulatory contexts that affect pharmacy-based care.

## Data Availability

The datasets used and/or analyzed during the current study are available from the corresponding author on reasonable request.

## Abbreviations

CCET: Community Collaboration and Engagement Team
CFIR: Consolidated Framework for Implementation Research
ERIC: Expert Recommendations for Implementing Change
IRLM: Implementation Research Logic Model
